# Larvicidal and Adulticidal Activity of Essential Oils from Four Cuban Plants against Three Mosquito Vector Species

**DOI:** 10.3390/plants12234009

**Published:** 2023-11-29

**Authors:** Jesús García-Díaz, Raimundo Nonato Picanço Souto, Julio César Escalona-Arranz, Ricardo Marcelo Dos Anjos Ferreira, Tiago Silva Da Costa, Rosalia González-Fernández, Yamile Heredia-Díaz, Idelsy Chil-Núñez, Jorge de la Vega, Lianet Monzote, Margareth Maria de Carvalho Queiroz, William N. Setzer

**Affiliations:** 1 Department of Basic-Biomedical Sciences, Faculty of Nursing-Health Technology, University of Medical Sciences, Carretera del Caney Km 2 1/2, Santiago de Cuba 90100, Cuba; jgadi1990@gmail.com; 2 Pharmacy Department, Natural and Exacts Sciences Faculty, Oriente University, Avenida Patricio Lumumba s/n, Santiago de Cuba 90500, Cuba; yherediad@gmail.com (Y.H.-D.); idelsychil@gmail.com (I.C.-N.); 3 Laboratory of Arthropoda, Department of Biology and Health Sciences, Universidade Federal do Amapá, Rodovia Jucelino Kubitschek, Km 02, Macapá 68902-280, AP, Brazil; nonatoiepa@hotmail.com (R.N.P.S.); rmaferreira@unifap.br (R.M.D.A.F.); 4 Department of Pharmaceutical Sciences, Faculty of Sciences, Universidad Católica del Norte, Angamos, Antofagasta 0610, Chile; 5 Laboratório de Arthropoda, Universidade Federal do Amapá, Campus Universitário Marco Zero do Equador, Rodovia Juscelino Kubitschek de Oliveira, Km 02, Bairro Zerão, Macapá 68902-280, AP, Brazil; tiago_sc@hotmail.com; 6 Programa de Pós-Graduação em Biodiversidade Tropical (PPGBIO), Universidade Federal do Amapá (UNIFAP), Macapá 68903-419, AP, Brazil; 7 Toxicology and Biomedicine Centre (TOXIMED), University of Medical Science, Autopista Nacional Km 1½ s/n, Santiago de Cuba 90400, Cuba; rosaliagonzalez.9110@gmail.com (R.G.-F.); jitodelavega@gmail.com (J.d.l.V.); 8 Department of Parasitology, Institute of Tropical Medicine “Pedro Kourí”, Apartado Postal No. 601, Marianao 13, La Habana 10400, Cuba; monzote@ipk.sld.cu; 9 Research Network Natural Products against Neglected Diseases (ResNetNPND), 48149 Münster, Germany; 10 Laboratory of Medical and Forensic Entomology, Instituto Oswaldo Cruz, IOC/FIOCRUZ, Rio de Janeiro 21040-360, RJ, Brazil; mmcqueiroz@ioc.fiocruz.br; 11 Programa de Pós-Graduação—Mestrado Profissional em Ciências Ambientais, Universidade de Vassouras—FUSVE, Av. Expedicionário Oswaldo de Almeida Ramos, 280, Vassouras 27700-000, RJ, Brazil; 12 Aromatic Plant Research Center, 230 N 1200 E, Suite 100, Lehi, UT 84043, USA; 13 Department of Chemistry, University of Alabama in Huntsville, Huntsville, AL 35899, USA

**Keywords:** essential oil, Lantana involucrata, Croton linearis, Zanthoxylum pistaciifolium, Ocimum sanctum var. cubensis, larvicidal, adulticidal

## Abstract

Mosquitoes are one of the main vectors of many important diseases and their degree of resistance to chemical insecticides has increased. Nowadays, it has become crucial to identify novel plant larvicides with an eco-friendly impact. The components of essential oils from *Croton linearis* Jacq. (EO-Cl), *Lantana involucrata* L. (EO-Li), *Ocimum sanctum* var. *cubensis* M. Gómez. (EO-Os), and *Zanthoxylum pistaciifolium* Griseb. (syn. *Zanthoxylum flavum* subsp. *pistaciifolium* (Griseb.) Reynel (EO-Zp) were determined using gas chromatography-mass spectrometry (GC–MS) analysis. Larvicidal and adulticidal bioassays against *Aedes aegypti*, *Anopheles albitarsis* and *Culex quinquefasciatus*, were performed according to the World Health Organization standard methods. A high diversity of compounds was identified in the four oils, with a total of 152 compounds (33–70 components). EO-Cl, EO-Li, and EO-Os were classified as active against both insect forms, larvae and adults. *Lantana involucrata* showed the best results, with LC_50_ values from 33.8 to 41.7 mg/L. In most of the cases, it was not possible to associate the main compounds with the measured activity, supporting the hypothesis about probable synergistic interactions among major and minor compounds. The results indicate EO-Cl, EO-Os, and EO-Li as good eco-friendly insecticides with potential.

## 1. Introduction

According to the World Health Organization (WHO), vector-borne diseases account for more than 17% of all infectious diseases, causing more than 700,000 deaths annually [1]. Dengue is the fastest spreading vector-borne disease, with an over 15-fold increase since 2000, and it affects over 129 countries. On the other hand, WHO reports that malaria is responsible for the highest global disease burden of all vector-borne diseases, causing approximately 405,000 deaths in 2018, most of them of children aged under 5 years. Other major vector-borne diseases include Chagas disease, Chikungunya, leishmaniasis, schistosomiasis, yellow fever, Zika, encephalitis, lymphatic filariasis, and other pathologies that cause a high rate of morbidity and mortality throughout the world [2,3].

During blood-feeding, mosquitoes (especially species of the genera *Aedes*, *Anopheles* and *Culex*) are the chief vectors involved in vector-borne diseases, transmitting infections to more than 700 million people. This prevalence has become a challenge for public health and has serious social and economic impacts, especially in tropical countries [2]. 

Unfortunately, this scenario has been exacerbated by many causes: rapid human population growth, limited funds for mosquito control programs, lack of awareness of environmental change and its consequences, and the adaptability of mosquito vectors. These causes are directly attributable to human actions and decisions, but one of the causes that has had the most significant impact in a short time is the phenomenon of resistance [4]. The widespread use of synthetic insecticides has led not only to mosquito resistance, but also to well-demonstrated adverse effects on the environment that contaminate soil, water, and air, generating secondary impacts on non-target populations, especially humans and other species from fauna and flora. Within this panorama, an increase in diseases transmitted by mosquitoes has been observed [3,5]. 

Consequently, efforts towards mosquito control continue to be an important strategy in preventing these mosquito-borne diseases. There are three main approaches described for mosquito control: (i) direct elimination in the adult phase, (ii) deterrent action against adults sucking blood, and the most explored, (iii) the reduction of larvae to decrease adult population density in the early stages of mosquito development [6]. Accordingly, the search for new natural insecticides with low toxicity toward humans and the environment has re-emerged in recent years, especially in those countries in which the regulatory requirements framework has been more flexible, such as Turkey, Uruguay, the United Arab Emirates, and Australia [7].

For thousands of years, plants and insects have evolved in parallel. This co-evolution has led to the development of chemical and physical defense mechanisms against insects, in which new plant characteristics have emerged to reduce enemy attacks. At the same time, plants also need the contribution of insects to carry out functions related to plant development, such as pollination. Therefore, plant metabolites are synthesized for both attraction and repellent/deterrent functions. This natural selectivity should be useful to develop some plant-based biopesticides. Unlike conventional commercial insecticides that are usually created based on a single active ingredient, plant-derived insecticides comprise botanical blends of secondary metabolites, which act in concert on both the behavioral and physiological processes of the target insects. Thus, the chance of insects developing resistance to such substances is more unlikely [8,9].

The development of bioinsecticides composed of botanical or plant-based compounds has been a perfect alternative due to their minimal hazardous effects on human health and the environment. From the various secondary metabolites of plants reported as insecticidal (larvicidal, adulticidal, and repellent), essential oils are perhaps the most explored. Plant essential oils are a complex mix of organic volatile compounds, in which terpenes and phenylpropanoids are the most common. Their characteristics give the essential oils the possibility to exhibit both attraction and repellent/deterrent functions. More than 3000 EOs from various plants have been analyzed thus far, and approximately 10% of them are commercially available as potential repellents and insecticides [10,11].

Cuba is a tropical island that boasts a rich flora used by the population to treat several disorders [12,13]. Tropical plants that grow under climatic conditions favoring microbial or insect attack have evolved a wide range of defense molecules [14]. In this sense, research into new eco-friendly insecticides from Cuban plants that grow abundantly and spontaneously throughout the island for the control of mosquitoes has barely been explored, missing affordable and cheap sources for obtaining biopesticides. In consequence, the present study aimed to assess the larvicidal and adulticidal effects of the essential oils obtained from four Cuban plants, *Croton linearis* Jacq. (EO-Cl), *Lantana involucrata* L. (EO-Li), *Ocimum sanctum* var. *cubensis* M. Gómez (EO-Os), and *Zanthoxylum flavum* subsp. *pistaciifolium* (Griseb.) Reynel (EO-Zp), against three of the most important mosquito vectors: *Aedes aegypti* (L.), *Anopheles albitarsis* Lynch Arribalzaga, and *Culex quinquefasciatus* Say.

## 2. Results

### 2.1. Yield and Chemical Composition of the Essential Oils 

The yields (expressed in v/w) of the EOs obtained by hydrodistillation from leaves of *C. linearis*, *L. involucrata*, *O. sanctum* var. *cubensis*, and *Z. pistaciifolium* were of 0.05%, 1.03%, 0.70%, and 1.60%, respectively. The GC-MS analyses of the four analyzed essential oils rendered 152 different compounds, as shown in Table 1. In the EO-Cl, 65 compounds were identified for 98.02% of the composition, with guaiol (8.27%) as the only compound with relative abundance over the 6%. For EO-Li, 65 compounds (96.94%) were identified, with α-bisabolol (15.33%), (*E*)-caryophyllene (13.04%), and 1,8-cineole (8.62%) as the most abundant. In the case of EO-Os, 31 compounds were identified from the total (95.38%), with eugenol (11.42%), (*E*)-caryophyllene (17.85%), and β-selinene (14.94%) as the major compounds. Finally, at least 67 compounds (98.41%) were identified in EO-Zp, with α-pinene (14.34%), limonene (7.24%), and linalool (7.81%) being the major ones.

### 2.2. Larvicidal Activity

The larvicidal activities of EO-Cl, EO-Li, EO-Zp, and the natural insecticide EO-Os are presented in Table 2. Three of the four tested essential oils were active (according to Cheng’s criteria [15]) against the three mosquito species studied, showing no selectivity between the strains tested (Table 2, H-test mosquitoes column).

Since EO-Os has been previously acknowledged as a natural larvicide [16], and its LC_50_ has been calculated and classified as active (according to Cheng’s criteria), their LC_50_ and LC_90_ were taken as internal references (LC_50_ < 70 mg/L and LC_90_ < 245 mg/L). In this context, EO-Li emerges as the most active essential oil (LC_50_ < 30 mg/L and LC_90_ < 190 mg/L), with a statistical difference (*p* < 0.05) regarding the other studied essential oils (Table 2, H-test oils column). In parallel, EO-Cl (LC_50_ < 65 mg/L and LC_90_ < 245 mg/L) showed a similar effect (*p* > 0.05) to the natural positive control, while EO-Zp was classified as not active (LC_50_ < 190 mg/L and LC_90_ < 400 mg/L) with statistical differences (*p* < 0.05) with respect to the other explored oils.

### 2.3. Adulticidal Activity

Based on the observed larvicidal effects (Table 2), the adulticidal activities of EO-Cl, EO-Li, and EO-Os were evaluated. The three essential oils showed activity at 24 h after 1 h essential oil exposure (Table 3). Mortality rates were directly proportional to concentration (concentration dependent) and statistically significant. During the hour of exposure to the essential oil, the mosquitoes showed restless movements, moving away from the area where the sample was placed, although several of them (mainly at the 200 mg/mL concentration) fell towards the bottom of the testing tube with symptoms of convulsion and paralysis. Once transferred to the holding tube, the highest number of deaths occurred within the first six hours, in which the mosquitoes initially showed excitement and then collapsed, with convulsive episodes, abnormal wagging and paralysis prior to death.

Against *Ae. aegypti*, EO-Cl was the most active, with LC_50_ = 79.94 mg/L (59.56–98.80) and LC_90_ = 158.87 mg/L (134.18–202.85), showing statistical differences (*p* < 0.05) with the natural reference insecticide (EO-Os) and the other essential oil tested (EO-Li). The EO-Li was numerically the second most active, but without statistical differences (*p* > 0.05) regarding EO-Os. The activity of these two was estimated in LC_50_ = 107.84 mg/L (88.17–128.60) and LC_90_ = 191.525 mg/L (163.94–240.61) for EO-Li, and LC_50_ = 119.85 mg/L (96.47–147.02) and LC_90_ = 226.67 mg/L (188.70–303.71) for EO-Os. In the other two mosquito species, no statistical differences (*p* > 0.05) were found between the essential oils tested. Nevertheless, the numeric values of LC_50_ and LC_90_ stated once again that EO-Cl was the most active against *An. albitarsis*, while against *Cx. quinquefasciatus*, EO-Cl and EO-Li showed almost the same statistical values as LC_50_, although with respect to LC_90_ value, EO-Cl showed stronger activity (Table 3).

## 3. Discussion

Plant essential oils are a complex mix of organic volatile compounds with repellent/deterrent functions. Approximately 10% of them are commercially available as potential repellents and insecticides, but these activities are determined under different biological models, laboratory protocols and conditions. Furthermore, it is well known that the chemical composition of essential oils can change depending on the conditions under which the plant is grown and harvested [17,18]. These variations generate changes in their activity profile. There are many examples in the literature: *Lippia gracilis* Schauer leaves collected in the same area of northeast Brazil, and carvacrol as the main compound classified as highly active by Cheng’s criteria (LC_50_ = 26.3 mg/L) [19], and as almost inactive (LC_50_ = 98.0 mg/L) two years later [20].

To counteract these limitations, in the present study we selected four essential-oil-producing plants whose composition had already been described by Cuban authors, but whose anti-mosquito activity had not been fully evaluated. At the same time, we selected plants that grow abundantly and spontaneously in Cuba, in such a way that they constitute (if they are very active) a source of raw material that is easy and economically viable to use. Among the studied plants, *O. sanctum* var. *cubensis* is more widely known, distributed, and used by the population, has the greatest number of reports as a bioinsecticide, and was therefore used as an internal control of activity.

With this purpose, four essential oil-producing plants belonging to four different botanical families were collected under similar conditions in terms of climatic and geographical location. *Zanthoxylum pistaciifolium*, an endemic plant from Cuba, appears to be the least studied, while *C. linearis* and *L. involucrata* (with limited distribution through the Caribbean area, but which grows abundantly and spontaneously in Cuba) have been studied previously. *Ocimum sanctum* var. *cubensis*, a member of the Lamiaceae family, was the species most studied, both from a chemical and a bioinsecticidal point of view, and therefore its essential oil was selected as the (natural) reference substance for the bioinsecticidal activity evaluated. For this selected natural standard (*O. sanctum* essential oil), recent studies confirm the ease and feasibility of its extensive cultivation, generating high levels of plant biomass and high levels of essential oil, making it a good candidate as a raw material for the extensive production of biopesticides [21]. Despite the number of hits found for the species under study, it was not possible to find a comprehensive evaluation of their activity against mosquitoes for any of them. Nevertheless, this study confirms the insecticidal property of Cuban plants and constitutes the relevance of this research in mosquito control, due to their known differences in biological activity with respect to the geographical location and season of plant collection.

The chemical composition analysis of the four essential oils in this investigation rendered 152 different compounds identified, with only nine common elements, in which three compounds were highlighted: elemene position isomers (*δ*, *β*, and *γ*), the widespread monoterpene linalool, and the sesquiterpene (*E*)-caryophyllene. This last compound ranked as one of the most abundant in two of the four oils studied. Then, certain similarities in their chemical composition with the previous studies conducted in the same collection area by Garcia et al. [22], Heredia et al. [23], and Chill-Núñez et al. [24], respectively, were found. In contrast, EO-Li shows differences from the report informed by Pino et al. [25], having been collected in a western region of Cuba.

Regarding the larvicidal activity, the selected positive control (natural insecticide: EO-Os) was classified as “active” according to the Cheng’s scale, in agreement with previous reports (LC_50_ = 67–69 mg/L) [26,27]. In this essential oil, the larvicidal activity of the main compound, (*E*)-caryophyllene (17.85%), remains controversial against *Ae. aegypti*. Huang et al. [28], Sarma et al. [29], and Hoi et al. [30] had reported (*E*)-caryophyllene to be active (LC_50_ = 56.34 mg/L, 49.78 mg/L and 65.92 mg/mL, respectively), while Doria et al. [31] found the compound to be inactive (LC_50_ = 1038 mg/L). The other two main compounds, *β*-selinene (14.94%) and eugenol (11.42%), have also been reported as having potent larvicidal effect against different species of mosquitoes [32,33,34]. Based on these precedents, it was expected that EO-Os would be classified as active, confirming its validity as the positive control.

EO-Cl was also classified as active within the same range of activity as the positive natural control (Table 2). In this case, there were too few reports related to its main compound, guaiol, as a larvicide, but it is a biting deterrent with similar activity to *N*,*N*-diethyl-*m*-toluamide (DEET) [34]. No other major compounds were identified in this essential oil, so the activity could be related to a synergistic effect of some of its minor components. *Croton* species that produce essential oils have been commonly considered active as larvicides. For example, *C. nepetaefolius* Baill essential oil (LC_50_ = 84 mg/L) and *C. zehntneri* Pax & K. Hoffm. essential oil (LC_50_ = 28 mg/L) against *Ae. aegypti* are two of them [35].

The most active larvicidal essential oil tested was EO-Li, with a LC_50_ range from 33 to 42 mg/L against the three mosquito species. Similar to EO-Os, (*E*)-caryophyllene (13.04%) was identified as one of the main compounds; therefore, this compound might contribute to the activity observed. Nevertheless, for the other two major compounds, *α*-bisabolol (15.33%) and 1,8-cineole (8.62%), no consistent activity has been published. 1,8-Cineole is reported to be active against larvae of *Cx. quinquefasciatus* (LC_50_ = 48.0 mg/mL) [36], while in another study the compound was found to be inactive against that mosquito species (LC_50_ > 250 mg/L) [37]. In contrast, against *Ae. aegypti*, different activity levels have been obtained (LC_50_ = 181.33 mg/mL [29] and LC_50_ = 74.91 ppm [38]). These facts make it difficult to point out any possible explanation for the activity observed based only on the activity of the main compounds. Thus, the high activity of the EO-Li must be attributable to a synergistic effect between major and minor components, as has been suggested for other essential oils [39]. Recent studies show that a binary mixture (1:1) between 1,8-cineole and *α*-pinene (compound representing 3.57% of the EO-Li) shows an appreciable synergistic effect, increasing the expected larvicidal activity estimated based on the LC_50_ of both pure compounds [29]. However, other studies report a non-significant contribution to this binary association [21].

On the other hand, EO-Zp is classified as not active (Cheng’s criteria), even when the main compounds, *α*-pinene (14.4%), linalool (7.81%) and limonene (7.24%), have been determined to show some larvicidal activities [15,29,39,40,41,42]. Once again, the tendency to associate the main compounds with the activity measured failed, giving place to another alternative explanation: an antagonistic effect among the main components by changing the rate of absorption, distribution, metabolism, or excretion, and/or a competition for the receptor or target site interaction. Experiments performed with binary mixtures (1:1) of *α*-pinene and limonene in the fourth-instar larvae of *Ae. aegypti* showed decreased larvicidal activity with respect to the pure compounds, demonstrating an antagonist relationship [29], but these results could not be confirmed in *Cx. quinquefasciatus* [37].

Regarding the selectivity of the essential oils for the three mosquito larvae, none of the extracts were able to discriminate among the species. Some papers in which two or more larva types were tested gave similar results. Govindarajan et al. did not find any different sensitivities in the evaluation of larvicidal activity when they essayed the essential oil from *O. basilicum* [43]. Nevertheless, the same author cited that the essential oil of *Mentha spicata* L. has selective toxicity faced with different mosquito larvae [44].

The other approach used to evaluate the insecticidal potential of the essential oils under study was adulticidal activity. In general, studies considering both vector control strategies used in this research (larvicidal and adulticidal) show that the larvicidal LC_50_ is reached at lower concentrations than the insecticidal LC_50_ ones [45,46]. This tendency was also observed in EO-Os, but especially by EO-Li. For EO-Cl, the LC_50_ values calculated were in the same range.

For EO-Os, eugenol (one of its main compounds) has been determined to be a good insecticide, with LC_50_ = 63.53 mg/L against *Ae. aegypti* 24 h post-treatment. This compound induced a considerable inhibition of acetylcholinesterase with an IC_50_ = 19.65 mM [47]. In in-silico studies, the inhibition of acetylcholinesterase by (*E*)-caryophyllene has been also suggested [48]. In addition, EO-Cl showed the best adulticide effect. In this essential oil, guaiol (8.27%) appears as the only compound in high concentration and has been reported to be a good insecticidal agent [49]. Similarly, EO-Li shows insecticidal effects, albeit slightly less actively. This essential oil is also classified as active against adult species of mosquitoes, an effect that can be attributed to the presence of 1,8-cineole (8.62%), one of its major components. The adulticidal activity has also been well recognized for this compound, with an LC_50_ value of 17.60 mg/L at 24 h exposure time [29]. Other authors have reported even lower LC_50_ values (LC_50_ = 0.74 mg/L) against *Ae. aegypti* adults [50].

For all tested essential oils, the activity of their main compounds exceeds the activity informed for the whole oil. In consequence, the activity observed in those essential oils might be interpreted as a result of the main compound activity “tuned” by its abundance in the oil and its biological interaction (synergism and antagonism) with the rest of the blend components. To find a reasonable argument for these findings, we correlated the available information on the activity of pure and binary mixtures of the most abundant terpenoids. Unfortunately, most studies have focused on monoterpene-type compounds rather than sesquiterpenes, which are the most abundant compounds in the species explored, even though most studies point to sesquiterpenes as the most active in their insect deterrent and anti-feeding action [51].

On the other hand, relatively recent studies experimentally demonstrated that some vectors are capable of metabolically detoxifying the major compound of an essential oil, but not the minor ones. In this way, the authors support, based on their experimental evidence, the possibility that the minor compounds in a sample acquire a more important role in the deterrent of or toxic effect on the insect than that which was granted until very recently [52]. These findings also contribute to the understanding of the higher toxicity of essential oils compared to their component terpenes, which highlight the use of essential oils as effective insecticides.

## 4. Materials and Methods

### 4.1. Plant Material and Extraction of Essential Oil

All plant material was collected in the morning (from 7:00 to 9:30 a.m.) from the Ecological Reserve Siboney-Juticí (natural habitat: lat. 19.958896, long. −75.734285), Santiago de Cuba province, in September 2021. A taxonomist from the Botany Department in the Eastern Centre of Ecosystems and Biodiversity (BIOECO, Santiago de Cuba, Cuba) authenticated the plants, and deposited them at the herbarium under the numbers EO-Cl—21659, EO-Li—21667, EO-Os—15955 and EO-Zp—21660. Essential oils were obtained through the hydro-distillation of fresh leaves from each of studied plants using a Clevenger apparatus for 3 h. The oil layer was separated from the aqueous phase using a separatory funnel. The resulting essential oils were dried with anhydrous sodium sulfate and stored in an amber-colored flask at 8 °C for chemical and biological assays.

### 4.2. Chemical Composition of the Essential Oils by GC/MS

The essential oils were analyzed in a Gas Chromatography Mega 2 series coupled to a mass spectrometer (GC/MS) Shimadzu GCMS-QP500 (Kyoto, Japan). A DB-5MS capillary column (Agilent Technologies, Santa Clara, CA, USA) of 30 m × 0.32 m and 0.25 μm thick film was used. The program temperature condition was 30 °C (3 min), with an increment of 4 °C/min until 250 °C, and it was then kept for 10 min at this temperature. The sample injection volume was 1 μL with a split ratio of 100:1, using helium as the carrier gas at a flow rate of 0.5 mL per minute. Both injector and detector temperatures were maintained at 290 °C. The percentage composition was calculated using the peak normalization method, assuming an equal detector response. A quadruple mass spectrometer analyzer with electron impact ionization at 70 eV was used to characterize the compounds, and identification was made by the comparison of Kovats retention indices and the mass spectra with the respective indices and reference mass spectra recorded in the NIST08 and FFNSC 1.3 library databases [53].

### 4.3. Mosquito Larvae Culture and Mosquito Adults

Larvae from *Ae. aegypti* came from eggs of the Rockefeller-CDC reference colony, while *Cx. quinquefasciatus* and *An. albitarsis* (variant s.l.) larvae were field-collected; identifications were carried out and first generation (F1) was used. All mosquitoes were maintained in the Medical Entomology Laboratory at the Amapá Federal University insectary at 27 ± 2 °C, 70 ± 10% relative humidity and a photoperiod of 12:12 h (light/dark) cycle, free from exposure to pathogens and insecticides according to the standards of the WHO [54]. Adults were maintained in cages (30 × 30 × 30 cm) and were continuously provided with 10% sucrose solution in a jar with a cotton wick. From day 5 onwards, the adults had access to blood meals from a rat placed in resting cages. Glass Petri dishes lined with filter paper soaked with 100 mL of de-chlorinated water were kept inside the cage for oviposition. The eggs thus obtained were immersed in larval trays containing de-chlorinated tap water for hatching. The larvae of *Ae. aegypti* and *Cx. quinquefasciatus* were fed on dog biscuits and *An. albitarsis* s.l. larvae were fed with Tetramin^®^ fish food. The third instar of *Ae. aegypti*, *An. albitarsis* s.l., and *Cx. quinquefasciatus* larvae were used for the larvicidal examination, and the adults were used for the adulticidal assay.

### 4.4. Larvicidal Assay

The larvicidal activity of the essential oils was assessed using an adaptation of the method recommended by the WHO [54]. Each essential oil was prepared in stock solutions to produce a range of five concentrations (20, 50, 100, 150, and 200 mg/L). Larvae were exposed to the five concentrations of essential oils from EO-Cl, EO-Li, EO-Os, and EO-Zp. In this experiment, EO-Os was considered to be a natural positive control according to international insecticide reports [16,55,56], while dimethyl sulfoxide (DMSO 1%) was used as a solvent control. The average value of the four replicates for each test concentration and species of mosquito larvae was noted. Batches of 10 active early third-instar larvae of *Ae. aegypti*, *An. albitarsis* s.l., and *Cx. quinquefasciatus* in 25 mL of distilled water were transferred into a glass beaker containing 74 mL of distilled water plus 1 mL of the experimental oil preparation, which allowed the essential oils to reach the desired test concentrations (20, 50, 100, 150, and 200 mg/L). This final solution of 100 mL was poured into a 200 mL capacity beaker. For solvent control, beakers with 99 mL of distilled water and 1 mL of DMSO were used. Mortality was recorded after 48 h of exposure, and the mortality percentage expressed the average value obtained from the four replicates. Mosquito larvae were recorded as dead when they showed discoloration, unnatural positions or rigor, if they were unable to rise to the surface, or when they showed the characteristic diving reaction when disturbing the water.

The criteria to characterize the efficacy of plant extracts such as mosquito larvicides vary from one author to another [57]. In this paper, we used the classification criteria established by Cheng and collaborators that established 100 mg/L as the breakpoint, taking into account the Median Lethal Concentration (LC_50_). In consequence, oils with LC_50_ > 100 mg/L were classified as not active, while LC_50_ < 100 mg/L were classified as active. Oils with LC_50_ values below 50 mg/L were considered highly active [15].

### 4.5. Adulticidal Assay

The adulticidal assay was performed following the instructions of the WHO methodology [58], with some minor modifications. Briefly, 25 female mosquitoes (2–5 days old glucose-fed, blood starved) were introduced in the holding tube for 1 h to acclimatize. After this time, the mosquitoes were gently transferred into another tube containing rectangular pieces (12 × 15 cm) of Whatman #1 filter paper soaked in 2.5 mL of different concentrations (20, 50, 100, 150, and 200 mg/L) of the essential oils to be tested. For the control group, Whatman #1 filter paper was embedded with ethanol 30% only. After 1 h exposure, mosquitoes were returned to the holding tube for another 24 h as a recovery period. A pad of cotton soaked with 10% glucose solution was placed on the mesh screen. The mortality of the mosquitoes was determined at the end of the 24-h recovery period. Each treatment was tested in triplicate. Three activity ranges were established to define the adulticidal capacity of essential oils: 1–49% was low, 50–69% moderate and 70–100% high [59].

### 4.6. Statistical Analysis

The percent mortality was corrected using Abbott’s formula. Mean larval mortality and the effect of concentrations were calculated using a probit regression analysis for calculating LC_50_ and LC_90_ at a 95% confidence limit, using the software SPSS 11.5 version package (SPSS, Chicago, IL, USA, 2007). The Kruskal–Wallis H test (*p* < 0.05) was used to compare the averages between mosquito species and essential oils. Differences between the treatments were determined using Dunn’s multiple comparisons test. Mean adult mortality was also estimated using the probit regression analysis for calculating LC_50_, LC_90_ and other statistics such as upper confidence limit (UCL) and lower confidence limit (LCL). GraphPad Prism 6.0 (San Diego, CA, USA) was used in this case. All results with *p* < 0.05 were considered statistically significant.

## 5. Conclusions

This study reported for the first time the evaluation of the larvicidal and adulticidal activity of four essential oils obtained from Cuban medicinal plants. The oils EO-Li, EO-Cl, and EO-Os showed a good effect against larvae and adults of three species of mosquitos: *Ae. aegypti*, *An. Albitarsis*, and *Cx. quinquefasciatus*. Chemical composition analyses of these oils revealed that they are composed of some bioactive molecules that may be responsible for the activity observed. In consequence, these essential oils could be good candidates to be developed as eco-friendly insecticides for mosquito vector control.

Despite these good results, further studies should be performed to better understand the mechanisms of action of these promising essential oils from Cuban plants and the contribution of their main components to the evaluated activity. In addition, to be considered for practical use, stable formulations of the essential oils should be investigated and field trials should be carried out.

## Figures and Tables

**Table 1:** Chemical constituents and their relative abundance of essential oils from leaves of *Croton linearis* (EO-Cl), *Lantana involucrata* (EO-Li), *Ocimum sanctum* var. *cubensis* (EO-Os), and *Zanthoxylum flavum* subsp. *pistaciifolium* (Griseb.) Reynel (EO-Zp).

Compounds	RI ^a^	RI ^b^	Relative Abundance (%)			
			EO-Cl	EO-Li	EO-Os	EO-Zp
(3*E*)-Hexen-1-ol	843	852		0.03		0.35
(3*Z*)-Hexen-1-ol	852	857				0.94
1-Hexanol	855	869				0.11
Tricyclene	920	926		0.36		
*α*-Pinene	929	939		3.57		14.34
*α*-Fenchene	949	953				0.89
Camphene	949	953		1.28		
Benzaldehyde	961	960		0.04		1.11
Sabinene	967	965	0.22	4.25		0.24
β-Pinene	978	975		0.7		1.5
1-Octen-3-ol	980	978		0.04		
(5*Z*)-Hepten-2-one	985	986		0.06		
Myrcene	991	991		0.57		3.61
*α*-Phellandrene	1005	1005				0.13
*δ*-Carene	1010	1009		0.15		
*α*-Terpinene	1017	1018		0.19		0.31
Limonene	1034	1031				7.24
1,8-Cineole	1038	1033	2.57	8.62		0.53
(*Z*)-*β*-Ocimene	1040	1041				2.76
(*E*)-*β*-Ocimene	1047	1050		0.3		3.28
*γ*-Terpinene	1059	1056	0.14	0.98		0.4
*cis*-Sabinene hydrate	1069	1068		0.35		
*cis*-Linalool oxide (furanoid)	1070	1069				0.26
Terpinolene	1082	1088		0.3		1.11
Linalool	1099	1093	0.04	1.93	0.8	7.81
(2*Z*)-Menthen-1-ol	1122	1110		0.07		
(4*E*,6*Z*)-*allo*-Ocimene	1126	1131		0.14		
2,6-Dimethyl-2,4,6-octatriene	1133	1149				7.6
*trans*-Pinocarveol	1137	1147		0.05		
(3*Z*)-Hexenyl 2-methylpropanoate	1143	1145				0.23
*trans*-Verbenol	1144	1148		0.14		
Camphor	1147	1150				0.16
Borneol	1171	1165		1.46		0.31
Terpinen-4-ol	1180	1180	0.54	0.98		1.23
Decan-3-one	1187	1188				0.68
Methyl salicylate	1189	1191				0.12
*α*-Terpineol	1197	1190	1.16	1.58		
*γ*-Terpineol	1201	1197				5.02
Nerol	1225	1226				0.16
(3*Z*)-Hexenyl 2-methylbutanoate	1232	1231				0.99
Hexyl 2-methylbutanoate	1237	1236				0.41
Geraniol	1252	1255				0.31
Geranial	1268	1270				0.12
*trans*-Verbenyl acetate	1278	1282	0.05			
Bornyl acetate	1285	1283	0.05			
Thymol	1294	1287	0.05			
Dihydrocarvyl acetate	1330	1305				0.29
*δ*-Elemene	1331	1327	0.05	2.7	2.63	1.21
*β*-Terpinyl acetate	1343	1359	0.09			
Eugenol	1352	1367		0.17	11.42	2.42
Cycloisosativene	1361	1362	0.18			
Isoledene	1367	1372	0.02			
*α*-Copaene	1370	1376		1.21	0.66	0.22
Longicyclene	1373	1374	0.5			
*β*-Bourbonene	1380	1388		0.9		
*β*-Elemene	1385	1392	4.33	0.67	0.51	5.39
Methyl eugenol	1387	1383			3.44	
(*E*)-Caryophyllene	1422	1423	1.28	13.04	17.85	3.06
*γ*-Elemene	1426	1425	1.4	0.88	2.19	0.35
*trans-α*-Bergamotene	1434	1436		1.74		
(*Z*)-*β*-Farnesene	1442	1439		0.43		
Isogermacrene D	1442	1442		0.58	0.63	
*ε*-Muurolene	1446	1446		0.66	0.53	
(*E*)-*β*-Farnesene	1452	1457		0.24	1.64	
*α*-Humulene	1454	1455	1.56	1.47	3.33	0.94
*allo*-Aromadendrene	1458	1461	0.53		1.52	
9-*epi*-(*E*)-Caryophyllene	1461	1467		0.81		
2-*epi*-(*E*)-*β*-Caryophyllene	1467	1469	0.44			
*β*-Acoradiene	1470	1472	0.27	0.94		
Selina-4,11-diene	1471	1476		0.3	0.27	0.58
*γ*-Gurjunene	1475	1479			6.64	0.46
Germacrene D	1478	1480		1.99		0.56
*α*-Amorphene	1482	1480	3.86			
*δ*-Selinene	1490	1485	3.27			
*trans-β*-Bergamotene	1492	1480		0.49		
*β*-Selinene	1493	1496			14.94	1.49
*α*-Zingiberene	1496	1495		0.86		
Bicyclogermacrene	1498	1500		0.35		
*α*-Muurolene	1499	1497	1.51	0.09		
*α*-Selinene	1500	1501			7.23	1.42
*ε*-Amorphene	1501	1497	0.61	0.29	0.43	
*δ*-Amorphene	1505	1505	1.97			
(*E*,*E*)-*α*-Farnesene	1507	1508		0.7		1.3
*β*-Bisabolene	1511	1510		0.94	5.37	
*γ*-Cadinene	1512	1513	2.07		0.32	0.1
Sesquicineole	1515	1516		1.02		
*δ*-Cadinene	1518	1518	0.45	1.23	0.18	0.63
*trans*-Cadina-1,4-diene	1520	1524	3.66			
*β*-Sesquiphellandrene	1521	1523		1.7	0.37	
Unidentified	1525	-	1.86			
7-*epi*-*α*-Selinene	1528	1526	3.07			
Selina-4(15),7(11)-diene	1535	1534	4.4			
*α*-Cadinene	1536	1536	0.1			
*trans-α*-Bisabolene	1538	1538		0.41		0.15
*α*-Calacorene	1540	1542	0.89			
*α*-Elemol	1548	1546	1.64	0.14		0.11
*cis*-Sesquisabinene hydrate	1549	1559		1.99		
*β*-Calacorene	1558	1555	1.09			
(*E*)-Nerolidol	1559	1559	0.19		0.20	0.15
(*Z*)-Nerolidol	1569	1568	0.79			
(3*Z*)-Hexenyl benzoate	1570	1571				0.75
Germacrene D-4-ol	1575	1574	1.63			
Spathulenol	1578	1578	3.93		3.07	
Caryophyllene oxide	1581	1582		7.78	0.98	0.4
Viridiflorol	1594	1590	0.63		0.7	0.59
Fokienol	1591	1596		0.53		
Guaiol	1602	1600	8.27			0.21
Humulene epoxide II	1607	1608		0.46		0.35
1,10-di-*epi*-Cubenol	1612	1614	2.66	0.45	0.25	
Copaborneol	1616	1613				1.23
1-*epi*-Cubenol	1617	1617	0.86			
*epi*-*γ*-Eudesmol	1624	1622			1.08	
Cubenol	1630	1627				4.53
*γ*-Eudesmol	1632	1632	0.1			0.14
11,11-Dimethyl-4,8-dimethylenebicyclo[7.2.0]undecan-3-ol	1636	1645		2.97		
Hinesol	1640	1640		0.42	0.8	
δ-Cadinol	1641	1640			2.58	0.83
Selin-11-en-4α-ol	1647	1650	0.3			
*α*-Cadinol	1658	1653	4.15	1.58	2.6	0.22
(*Z*)-Nerolidyl acetate	1662	1665		0.16		2.12
Intermedeol	1664	1666	0.77	0.18	0.22	1.09
3,10(14)-Guaiadien-11-ol	1672	1672	4.75			
(9*Z*)-Tetradecen-1-ol	1676	1667				0.29
4(15),5,10(14)-Germacratrien-1α-ol	1680	1678	2.8			
14-Hydroxy-9-*epi*-(*E*)-β-caryophyllene	1683	1682	2.08			
*α*-Bisabolol	1684	1684		15.33		0.11
*epi*-*α*-Bisabolol	1688	1688	0.62			
4(15),7-Eudesmadien-1β-ol	1699	1690	5.19			
7-Hydroxyeudesm-4-en-6-one	1708	1703	2.94			
(*Z,Z*)-Farnesol	1712	1713				0.26
Eudesma-4,11-dien-2-ol	1714	1714	0.7			
Cedrenone	1722	1722	0.21			
Isolongifolol	1729	1723	0.96			
*γ*-Costol	1733	1732	0.3			
9(*E*)-Humulen-2,6-dione	1739	1739	1.15			
Drimenol	1746	1745	3.11			
*β*-Bisabolen-12-ol	1762	1753	0.68			
*α*(*E*)-Atlantone	1768	1773	0.6			
*n*-Tetradecanoic acid	1769	1770				0.2
14-Oxy-α-muurolene	1779	1776	0.87			
(*E*)-Isovalencenol	1783	1783	0.36			
*epi*-Cryptomeridiol	1791	1790	0.18			
14-Hydroxy-δ-cadinene	1803	1803	0.32			
Total (%)			98.93	96.94	96.09	99.05

RI ^a^: Kovats retention index, RI ^b^: reported retention indices under similar experimental conditions.

**Table 2:** Larvicidal activity (concentrations required to kill 50% (LC_50_) and 90% (LC_90_) of *Croton linearis* (EO-Cl), *Lantana involucrata* (EO-Li), *Ocimum sanctum* var. *cubensis* (EO-Os) and *Zanthoxylum flavum* subsp. *pistaciifolium* (Griseb.) Reynel (EO-Zp) essential oils on the third-stage larvae of *Aedes aegypti*, *Anopheles albitarsis* and *Culex quinquefasciatus*.

Plant/Mosquito	LC_50_ (mg/L) (95% CI)	LC_90_ (mg/L) (95% CI)	H-Test Mosquitoes	H-Test Oils
EO-Cl				
*Aedes aegypti*	64.63 (49.52–81.31)	243.82 (157.55–377.36)	0.69 (ns)	12.88 ^b^
*Anopheles albitarsis*	63.54 (48.67–79.84)	237.04 (149.26–376.45)		12.90 ^b^
*Culex quinquefasciatus*	64.63 (49.52–81.81)	243.82 (157.55–377.36)		12.64 ^b^
EO-Li				
*Aedes aegypti*	33.79 (24.99–45.71)	182.77 (135.16–247.16)	2.22 (ns)	12.88 ^a^
*Anopheles albitarsis*	38.41 (25.91–50.42)	179.29 (124.09–259.05)		12.90 ^a^
*Culex quinquefasciatus*	41.72 (29.14–53.94)	187.37 (149.48–275.61)		12.64 ^a^
EO-Zp				
*Aedes aegypti*	182.08 (157.18–230.45)	399.01 (291.80–781.55)	0.69 (ns)	12.88 ^c^
*Anopheles albitarsis*	182.08 (157.18–230.45)	399.01(291.80–781.55)		12.90 ^c^
*Culex quinquefasciatus*	185.75 (160.64–235.97)	396.33 (290.81–779.69)		12.64 ^c^
EO-Os (natural insecticide)				
*Aedes aegypti*	68.98 (53.92–85.94)	243.96 (156.57–380.14)	0.69 (ns)	12.88 ^b^
*Anopheles albitarsis*	68.98 (53.92–85.94)	243.96 (156.57–380.14)		12.90 ^b^
*Culex quinquefasciatus*	67.97 (53.41–84.23)	234.23 (148.07–370.54)		12.64 ^b^

DMSO: dimethyl sulfoxide. H-test = Kruskal–Wallis H test. Different letters in superscripts mean Dunn’s statistical differences (*p* < 0.05). ns: no statistical differences.

**Table 3:** Adulticidal effects (estimated values of LC_50_ and LC_90_) of *Croton linearis* (EO-Cl), *Lantana involucrata* (EO-Li), and *Ocimum sanctum* var. *cubensis* (EO-Os) essential oils against three mosquito species measured 24 h after 1 h exposure.

Plant/Mosquito	LC_50_ (mg/L) (95% CI)	LC_90_ (mg/L) (95% CI)	H-Test Mosquitoes	H-Test Oils
EO-Cl				
*Aedes aegypti*	79.94 (59.56–98.80)	158.87 (134.18–202.85)	2.76 (ns)	6.49 ^a^
*Anopheles albitarsis*	69.76 (46.70–89.26)	153.53 (127.98–200.66)		5.40 (ns)
*Culex quinquefasciatus*	78.63 (54.66–99.54)	170.91 (142.57–224.10)		5.40 (ns)
EO-Li				
*Aedes aegypti*	107.84 (88.17–128.60) ^b^	191.53 (163.94–240.61)	6.49 *	6.49 ^a,b^
*Anopheles albitarsis*	97.35 (75.81–118.75) ^a,b^	188.23 (159.02–241.98)		5.40 (ns)
*Culex quinquefasciatus*	77.12 (49.34–100.04) ^a^	181.67 (149.59–245.68)		5.40 (ns)
EO-Os (natural insecticide)				
*Aedes aegypti*	119.85 (96.47–147.02)	226.67 (188.70–303.71)	4.62 (ns)	6.49 ^b^
*Anopheles albitarsis*	92.97 (65.91–118.12)	205.86 (169.08–282.33)		5.40 (ns)
*Culex quinquefasciatus*	101.59 (75.52–127.78)	215.44 (177.13–295.58)		5.40 (ns)

ns: no statistical differences; * selectivity between mosquito strains (*p* < 0.05). Different letters in superscripts mean Dunn’s statistical differences (*p* < 0.05).

## Data Availability

All data are available in the article.
